# Exploring the presentation of REDs in ultra endurance sport: a review

**DOI:** 10.1186/s40337-025-01381-0

**Published:** 2025-09-26

**Authors:** Jill Colangelo, Alexander Smith, Keely Henninger, Anna Buadze, Michael Liebrenz

**Affiliations:** 1 Department of Forensic Psychiatry, University of Bern, Falkenplatz 18, Bern, 3012 Switzerland; 2 Oregon Health and Sciences University School of Medicine Portland, Portland, OR USA; 3 Department of Psychiatry, Psychotherapy and Psychosomatics, Psychiatric Hospital, University of Zurich, Zurich, Switzerland

**Keywords:** REDs, Ultra endurance, Sports psychiatry, Mental health, Disordered eating

## Abstract

**Introduction:**

Ultra-endurance sports are characterized by prolonged durations of activity, high energy expenditure, and unique physiological and psychological demands. These factors could exacerbate risks for Relative Energy Deficiency in Sport (REDs) and the related conditions of low energy availability (LEA), eating disorders (ED), and disordered eating (DE), amongst others. However, presently there is a lack of information about these topics in ultra-endurance sports.

**Methods:**

To explore risk factors, lived experiences, and research gaps, we conducted a narrative review of studies exploring REDs in ultra-endurance sports. A keyword search was performed in Scopus and PubMed, with supplemental searches of reference lists and Google Scholar. Both quantitative and qualitative studies were included to capture the scope of available evidence. Following selection and extraction, key quantitative findings were summarized and qualitative research was thematically analyzed.

**Results:**

Sixteen studies (*n* = 10 quantitative; *n* = 6 qualitative/case reports) were included in the results. Quantitative studies highlighted the presence of REDs, LEA, and DE/ED among ultra endurance athletes, with one studying showing that up to 65% of athletes may be at-risk. Qualitative studies provided insights into psychological distress, body image concerns, intentional caloric restriction, and the impact of sociocultural pressures on fueling behaviors in athletes.

**Conclusions:**

REDs and related conditions pose risks for ultra-endurance athletes, with the combination of high-volume training and psychological drivers likely increasing susceptibility to energy imbalances. Nevertheless, the REDs model may inadequately address underlying psychological factors contributing to LEA and could require integration with ED-based protocols for improved treatment outcomes in ultra-endurance sports and elsewhere.

## Abstract (plain language summary)

Ultra-endurance sports involve high training volumes and extreme energy demands. These factors may increase risks for Relative Energy Deficiency in Sport (REDs), which is a condition where athletes do not sufficiently fuel to support their activity level, potentially leading to serious health issues. This review explored existing quantitative and qualitative research on REDs and related problems like low energy availability, disordered eating, and eating disorders in ultra-endurance athletes. Findings showed that up to 65% of ultra-endurance athletes may be at risk for REDs. Psychological factors were linked to restrictive eating and overtraining. Despite this, REDs is often treated as a physical problem, with less attention paid to mental health. This review suggests that treatment should address both physical and psychological needs, especially for athletes who restrict food intentionally. It also highlights the need for further research on recreational, male, and gender-diverse athletes, who remain overlooked despite being at-risk.

## Background

### Ultra-endurance sports and their physiological and psychological demands

Generally, ultra-endurance sports encompass events lasting six hours or more and are characterized by unique physiological, psychological, and sociocultural factors [1]. Ultra-endurance sports typically involve high volume training with co-occurring high energy expenditure; competitions last for extended durations without distinct rest periods, incurring substantial energy demands and subsequent deficits in energy intake [2, 3]. Daily macronutrient needs can exceed 12 g of carbohydrate per kilogram of bodyweight and 1.20–2.00 g of protein per kilogram of bodyweight [4]. Caloric demands during racing can vary from ~ 3000 to > 8000 calories per day depending on the duration and climate [4]. Nutritional requirements include separate considerations for daily training, pre-race preparation, intra-race fueling, and post-race replenishment [4, 5]. Recovery and replenishment of calories intended to restore energy balance for ultra-endurance athletes must be considered in daily training and can take hours to several days to correct after race efforts [6]. Research indicates that injury, illness, and other negative physiological outcomes might result from remaining in energy deficit [6], which frequently affects ultra-endurance athletes, either through intentional or unintentional behaviors [7]. Moreover, ultra-endurance athletes may be subject to increased body awareness, vanity, and/or poor body image, with concomitant influences on nutritional strategies [8–10].

Another unique aspect of ultra-endurance sports is that both recreational and professional athletes will commonly be training at volumes (i.e., the number of hours per week) normally associated with elite-level expectations [5]. There is a wide spectrum of training protocols for ultra-endurance sports, which may vary according to the sport and the pace of the athlete. For example, ultra-endurance athletes may run fewer kilometers per week than elite competitors, but they may be spending equal or more time training as they possibly have a slower pace. In other sports, such as triathlon, which require training in three separate disciplines, the time recreational participants spend training may also be comparable to elites due to a relatively slower pace. Elsewhere, evidence indicates that recreational Ironman triathletes and endurance cyclists may train thirteen hours per week or more, which is comparable to elite volumes [11, 12].

Recreational athletes have the same need to remain in energy balance; however, while elite athletes are generally supported by multiple ancillary professionals (e.g., coaches or nutritionists), recreational ultra-endurance athletes might rely upon self-knowledge, personal research, or community-based information, which could be inadequate for sustaining high-volume training healthfully [7]. As first-hand accounts demonstrate, recreational athletes might also participate in UES for deliberate weight control and other related motivations, since the idea that lowering weight may lead to better performance persists [13, 14].

### Ultra-endurance sports, relative energy deficiency in sport, and related conditions

Owing to the distinctive demands from the volumes of training and energy availability associated with these sports, ultra-endurance athletes might be at greater risk of chronic energy imbalances [15, 16]. In this regard, low energy availability (LEA) is the state in which athletes can become compromised (physically and psychologically) due to inadequate energy intake, as well as overwhelming energy expenditure [17]. LEA can cause Relative Energy Deficiency in Sport (REDs), a syndrome affecting all genders through a constellation of symptoms, such as endocrinological disturbances, compromised bone health, and musculoskeletal injury. In certain studies, REDs has come to supersede a previous term, Female Athlete Triad (hereafter the Triad), which insufficiently represented gendered dynamics and the symptomatological and clinical complexities [18]. That said, academic literature does not universally reflect these terminological changes and therefore, studies on the Triad are not altogether obsolete for examining energy imbalances and their related effects.

Correspondingly, research has demonstrated an experiential and symptomatological overlap between REDs and eating disorders (ED) and disordered eating (DE) [19, 20]. Specifically, ED are defined as mental illnesses involving abnormal eating behavior that fulfills key diagnostic criteria in the ICD-11/DSM V and causes distress [20, 21]. Meanwhile, DE encompasses abnormal eating behavior that may not completely meet ICD-11/DSM-V criteria, but does provoke psychological distress, increased risk for potential injury, and/or psychological strain [20]. It is important to note that ED are recognized as mental disorders in psychiatric diagnostic manuals, whereas REDs is a syndrome that can generally be used to describe the consequences of LEA [19, 21]. Conversely, LEA could emerge from the pursuit of increased physical activity, leading to large energy deficits and/or from restricting caloric intake (either intentionally or unintentionally) [22].

Similarly, exercise dependence (EXD) is not currently recognized as a mental disorder by either the DSM-V or the ICD-11, but it is a condition that can arise when an athlete develops an unhealthy relationship with sports that they are unable to modulate [22]. This does not account for all cases of LEA leading to REDs, but links between REDs and LEA could potentially emerge if the athlete is unable to adjust energy intake as they increase training volumes [15, 22]. EXD has not been formally classified due to variances in the interpretation of risk among assessments and debates on the definitions, etiology, and pathophysiology of behavioral addictions [23, 24]. Nonetheless, EXD can be considered *primary* when the unhealthy relationship develops in the absence of ED and *secondary* when it arises in correlation and coordination with ED [25]. Since EXD vulnerabilities in ultra-endurance sports have been documented, concomitant risks for LEA and ED might be high [22, 26].

Conversely, REDs can stem from unintentional underfueling due to inadequate knowledge, food intolerances/allergies, socioeconomic factors, or other reasons unrelated to weight control [27]. With this, REDs indicators may also present in a way that could be interpreted as symptomatic of other psychiatric disorders, such as depression and/or anxiety, conceivably overshadowing the need for mental health treatment in lieu of dietary adjustments [28]. Specifically, REDs treatment generally involves education and efforts to maintain energy balance, while therapeutic mechanisms for ED include psychiatric support as a primary intervention [19]. Nevertheless, best practice guidelines for REDs recovery protocols include assessment by an ED-informed mental healthcare practitioner [17]. Ultimately, whether intentional or unintentional, all forms of underfueling could engender serious pathology if unaddressed [22].

### The current review

REDs and corresponding LEA can pose concomitant health risks for athletes [29]. ED and DE have been associated with athletes of all ages, genders, and competitive levels [21, 30–32]. As mentioned, ultra-endurance sports could increase vulnerabilities for these issues since many athletes are dissatisfied with their weight, driven by a common notion that “lighter is faster” [33, 34]. Though this axiom is predicated on principles of mass and velocity, current research does not holistically support the hypothesis that decreasing body weight will have a positive or lasting effect on performance [35–38]. Notably, the pursuit of weight loss for competitive advantage often requires professional guidance, is usually prescribed for the very short term, and may lead to chronic LEA [36–38]. Ultra-endurance athletes can have heightened vulnerabilities for LEA as they often compete in races longer than six hours with higher training volumes relative to other sports, and many are falling short of the recommended fueling requirements [1, 22].

Nevertheless, it remains unknown whether ultra-endurance sports would also incur risk commensurate with other sports or if additional factors characteristic of these sports would increase susceptibilities for REDs and the related conditions of ED and DE. Against this background, we aimed to examine the current knowledge base on REDs in ultra-endurance sporting contexts. Specifically, the review sought to explore research related to ultra-endurance athletes, with particular attention to how the unique demands of these sports and their training load may interact to increase risk for LEA and related physiological and psychological issues.

## Methods

### Eligibility criteria

We conducted a narrative review of relevant literature discussing REDs and the related conditions of ED, and/or DE in ultra-endurance sports. Studies that did not contain athlete samples training for or competing in ultra-endurance distance events (i.e., those lasting six hours or more) and those that not explicitly indicating training consistent with UES (i.e., “runners” without further detail) were excluded. Non-primary sources (i.e., editorials, commentaries, and book chapters) were ineligible, as was literature that was not in English. To capture the full scope of extant sources, no other methodological or publication date parameters were applied; consequently, case reports that reported empirical results were eligible. Quantitative research designs were included to describe risk trends across ultra-endurance athletes. Likewise, as these conditions are typically assessed in the context of personal, sociocultural, and environmental concerns [20], the review also incorporated qualitative research to gain insights into athlete attitudes and motivations.

### Search strategy, extraction and analysis

One member of the research team searched Scopus and PubMed for studies available in English, using the search terms “eating disorder”, “disordered eating”, “REDs”, “female athlete triad”, “low energy availability”, “ultra endurance”, “ultra cycling”, “triathlon”, “ultramarathon”, “ultra swimming”. Subsequently, Google Scholar was searched to identify additional research and pertinent studies were also incorporated from eligible reference lists. Following this search, two members of the team screened titles and abstracts to ensure applicability within the present analysis, with consultation with a third member where necessary.

For quantitative studies, data was extracted to an Excel spreadsheet, including information related to the methodological type, type of assessment(/s) (where appropriate), study subjects, year of the study, and authors. To explore qualitative findings, we utilized the JBI Global Manual for Evidence Synthesis, displaying the Population (i.e., athlete groups), Phenomena of Interest (i.e., experiences related to REDs, LEA, disordered eating, and psychological distress), and Context (i.e., type of sport/sporting environment) (PICo) for each study [39]. Where appropriate, illustrative participant quotes were extracted only when presented as part of the original author’s published thematic analysis. The selection of quotes for inclusion was guided by their relevance to the central phenomena of interest (e.g., REDs, disordered eating, LEA) and their utility in illustrating key patterns or divergences grouped across the studies; all illustrative excerpts are credited to their respective sources in the results.

## Results

### Overview of results

A total of *n* = 16 studies were identified discussing DE/ED and/or REDs/the Triad in ultra-endurance contexts; *n* = 10 (62.5%) studies reported quantitative data and *n* = 6 (37.5%) studies presented qualitative data or were case reports. Of the quantitative research, *n* = 7 (43.8%) studies incorporated psychometric assessments and *n* = 5 (31.3%) relied on biometric data to uncover risk. Of the qualitative research, *n* = 3 utilized semi-structured interviews, *n* = 1 was a case report, *n* = 1 was a multiple case report using integrated methods, and *n* = 1 was a narrative analysis.

### Quantitative data in ultra-endurance sports

Table 1 summarizes the quantitative research, outlining the year of publication, number and description of study subjects, adopted methodology, and key findings.

**Table 1:** Quantitative research summary

Author	Date	Population, sex, age*	Training Volume**	Instruments	Relevant findings
Micklesfield, Hugo, et al. [40]	2007	*n* = 613 female runners (*n* = 276 ultramarathon runners) (*M* age: 39; range: 22–61)	1–3 h per week (5.1%); 4–6 (34.4%); 7–9 (44.6%); >10 (15.9%)	Self-loathing subscale (SLSS), demographic information and health/injury history, as well as menstrual function and substance use, etc.	61% of all women in study had menstrual irregularity, 21% ultrarunners had bone stress injury, women with menstrual irregularity had higher score on SLSS (SLSS; *p* < 0.01) indicating risk for the Triad. Significant differences between general runners and ultramarathon runners in having components of the Triad; 40% of ultramarathon runners reported at least one component of the Triad
Folscher, Grant, Fletcher et al. [41]	2015	*n* = 306 female ultrarunners (*M* age: 39; min/max: 21–65)	Mean hrs per week in peak season: 8.66 Range: 1.5–20	LEAF-Q, Female Athlete Screening Tool (FAST), additional questions	44.1% were at-risk for the Triad, yet 92.5% of the sample had not heard of the Triad previously. 26.8% were at-risk for subclinical ED, and 5.2% were at-risk for ED
Papadopoulou, Xyla, et al. [42]	2017	*n* = 24 male open water swimmers (12 were of a younger age: 26.3 ± 4.9 years; 12 were of an older age: 45.8 ± 9.7 years)	Training frequency per week: 5.71 ± 2.2, training volume per day: 15.6 ± 5.8 km	Dietary intake, energy intake/expenditure/balance, height, weight and Body Mass Index (BMI)	Severe malnutrition was evident in both groups: negative energy balance before the event (ranging between − 435.4 kcal - −458.1 kcal). All athletes were underfueled for ultra-endurance sports during competition and experienced resulting negative effects, including certain poorer performance outcomes
Lane, Hackney, et al. [16]	2019	*n* = 108 male endurance athletes (age: 38.6 ± 13.8)	Hours of exercise per week: 12.2 ± 5.4	Descriptive information about diet, exercise, and injuries	47.2% were found to be at-risk for LEA
Torstveit, Fahrenholtz, et al. [43]	2019	*n* = 53 well-trained male cyclists, triathletes, and long-distance runners (age: 35.3 ± 8.3)	Hours of exercise per week: 9.5 ± 3.4	Exercise Dependence Scale (EXDS), ED Examination Questionnaire (EDE-Q), Resting Metabolic Rate (RMR), body composition, caloric intake, blood measures, etc.	Higher EXDS scores were identified in those who were underfueling (*p* < 0.01). The EXDS total values were positively correlated with EDE-Q scores (*r* = 0.41, *p* < 0.05), as well as the subscale scores for restraint eating (*r* = 0.34, *p* < 0.05) and weight concerns (*r* = 0.35, *p* < 0.05). Higher cortisol was evident in those with higher EXDS scores (*r* = 0.38, *p* < 0.01)
Stenqvist, Torstveit, et al. [44]	2020	*n* = 22 male endurance cyclists (age: 33.5 ± 6.6 years)	Exercise hours per year: 395 ± 171	RMR, X-ray absorptiometry (DXA), blood sample, caloric intake, peak power, peak oxygen uptake, (VO_2peak_), functional threshold power, testosterone, cortisol, thyroid hormone, etc.	Performance improvements were observed with negative health consequences; REDs indicators showed unfavorable changes. There was a decrease in RMR (3.0%, *p* = 0.01) and thyroid hormone (T_3_) (4.8%, *p* = 0.008), and an increase in cortisol (12.9%, *p* = 0.02)
Fahrenholtz, Melin, et al. [15]	2022	*n* = 202 female endurance athletes (*M* age: 25; range: 21–30)	Training hours per month: 48.2 ± 19.7	LEAF-Q, Exercise Addiction Inventory (EAI), EDE-Q, self-developed ED questions, food tolerance questions, menstrual function questions BMI	65% were at-risk for LEA, 23% were at-risk for EXD, 21% were at-risk for DE behaviours
Høeg, Olsen, et al. [45]	2022	*n =* 123 ultramarathon runners *(n* = 83 male; *M* age: 46.2 (SD 10.3)); *n* = 40 female (*M* age: 41.8 (SD 7.6))	Running time (h/wk) men: 10 (SD 3.1) Running time (h/wk) women: 12.7 (SD 3.2)	Dual-energy x-ray absorptiometry scans, sex hormones, vitamin D, ferritin, questions derived from the Female Athlete Triad Screening Questionnaire, the Triad Consensus Panel Screening Questionnaire, and the dietary restraint and pathologic behavior sections of the EDE-Q, etc.	Elevated risk for DE was identified in 44.5% of men and 62.5% of women. History of bone stress injury was present in 37.5% of women and 20.5% of men. DXA < 1.0 in 16.7% women and 30.1% of men. Low BMI was evident in 15% women and 0% men. 61.1% women and 29.2% men were at moderate risk and 5.6% of both men and women were at high risk using Triad Cumulative Risk Assessment
Reno-Smith, Pritchett, Henninger [34]	2024	*n* = 1021 masters trail/ultra runners (*n* = 457 male/*n* = 564 female) (age range: 41–65)	Not reported	Disordered Eating Screen for Athletes (DESA-6), Exercise Dependence Scale (EDS-21), and additional questions	79% of males and 80% of females exhibited indicators of EXD. 71.4% of participants were dissatisfied with their weight. 46% of females and 32% of males were at-risk for DE
Henninger, Pritchett, et al. [22]	2024	*n* = 1899 trail/ultra runners (*n* = 510 male/*n* = 1445 female) (age range: 18–40)	54% of participants ran 31–60 miles per week and 10% of participants ran >60 miles per week	LEAF-Q, DESA-6, EDS-21, and additional questions	43% of the sample were at-risk for LEA, 43% were at-risk for DE, and 87.3% exhibited indicators of EXD

* Athlete groupings and age data as reported in the study ** volume data as reported in the study

#### Risk for DE, ED, and/or LEA

Of the quantitative studies included, 43.8% (*n* = 7) specifically explored vulnerabilities for ED, DE, and/or LEA [16, 22, 34, 40, 41, 45]. Biomarkers associated with REDs risks were discussed in *n =* 1 study (6.3%) [44], while *n* = 1 study (6.3%) discussed EXD risks as a precursor to ED, DE, and/or LEA [43]. Additionally, another study (6.3%) explored malnutrition in the sample by measuring nutritional intake both before and during an ultra-endurance event using self-report questions and approximate energy expenditure [42]. Notably, methodological designs were heterogeneous in their measurement of LEA, with some (*n* = 6; 37.5%) relying on self-report psychometric questionnaires and others (*n* = 4; 25%) using mathematical computation of LEA through biometric assessment performed in a laboratory setting [44]. Additionally, there was great heterogeneity in the types of psychometric tests adopted in quantitative work on REDs in ultra-endurance sports, alongside the supplemental demographic questions that underpinned these risk assessments.

#### Psychometric assessment of risk of ED, DE, EXD, and LEA

Several studies (*n* = 7; 43.8%) used psychometric assessments to measure risk of ED, DE, EXD and LEA. In an investigation of 306 female ultrarunners, 26.8% were found to be at-risk for subclinical ED and 5.2% were at- risk for ED using the Low Energy Availability in Females Questionnaire (LEAF-Q) and Female Athlete Screening Tool (FAST) [41]. Another study used the LEAF-Q, Exercise Addiction Inventory (EAI), Eating Disorder Examination Questionnaire (EDE-Q) with 202 female ultra-endurance athletes, finding that 65% were at-risk for LEA, 23% were at-risk for EXD, and 21% were at-risk for DE [27]. Furthermore, in a sample of 1899 male and female trail and ultra runners, 43% were at-risk for LEA, 43% were at-risk for DE, and 87.3% had symptoms related to EXD per the LEAF-Q, the Disordered Eating Screen for Athletes (DESA-6), and the Exercise Dependence Scale-21 (EDS-21) [22]. In a separate study, the overall risk for DE was found to be 88.2% in 1021 masters trail and ultra runners [34]. Specifically, this study used the DESA-6 and EDS-21 to ascertain that 46% of females and 32% of males exhibited risks for DE, and 80% of females and 79% of males presented with indicators of EXD [34].

Elsewhere, researchers investigated whether ED risks could be established by examining the close relationship between EXD and REDs in the presence of stress hormone, using the Exercise Dependence Scale (EXDS), ED Examination Questionnaire (EDE-Q), Resting Metabolic Rate (RMR) and other biomarkers [43]. This sample of 53 well-trained male cyclists found that EXD positively correlated with ED risk, and higher scores on the EXDS subscale were correlated with higher cortisol [43]. Separately, the Self-Loathing Subscape (SLSS) was used with other biometric data to uncover risk for the Triad in 276 female ultramarathon runners [40]. Women in this study with menstrual irregularity (61%) had higher SLSS scores, indicating greater vulnerabilities for the Triad [40]. Finally, in a sample of 123 ultrarunners, the Triad Cumulative Risk Assessment found that 61.1% of women and 29.2% of men were at moderate risk, and 5.6% of both women and men were at high risk for indicators of the Triad [45].

#### Biometric, computational assessment of risk

Another group (*n* = 4; 25%) of designs captured in our review utilized mathematical computation to assess risk in athletes. In a study of 24 male open water swimmers (12 younger, mean age: 26.3 years; 12 older, mean age: 45.8 years) were found to be malnourished generally, in negative energy balance before event (− 435.4 kcal - −458.1 kcal), and consuming less than the recommended intake of both macro and micronutrients during competition [42]. Despite relative differences in energy needs and intake between the groups, both older and younger athletes reported inadequate consumption of overall calories for the energy demands of the competition [42]. In a study of *n =* 108 male endurance athletes, diet and training records were analyzed to ascertain energy balance, finding that LEA risk was evident in 47.2% of participants [16].

One study used multiple biometric assessments, such as RMR, X-ray absorptiometry (DXA), blood sample, caloric intake, peak power, peak oxygen uptake, (VO_2peak_), functional threshold power, testosterone, cortisol, and thyroid hormone to understand the effects of a four-week period of intense training on biomarkers associated with REDs in 22 ultra endurance cyclists [44]. Negative health consequences were apparent when analyzing biomarkers associated with REDs including a decrease in RMR (3.0%, *p* = 0.01) and thyroid hormone (T_3_) (4.8%, *p* = 0.01), plus an increase in cortisol (12.9%, *p* = 0.02) [38]. Finally, again in their study of *n =* 123 ultrarunners, researchers adopted DXA to find that 16.7% women and 30.1% of men had Z scores of < -1.0, which would indicate compromised bone mineral density, osteopenia, and possible evidence of the Triad [45].

### Qualitative data and case reports

The following table (Table 2) summarizes the *n* = 6 (37.5%) qualitative studies in our review, displaying relevant information. Though all studies focused on athletes training at ultra-endurance volume and involved discussions of REDs, DE, and/or ED, they remained heterogeneous in their methodology, context, and the phenomena of interest. Nevertheless, overlapping themes and commentary about REDs were ascertained from the athletes’ experiences and were extracted from the studies.

**Table 2:** Qualitative research summary using PICo summary

Authors	Date	Population*	Phenomena of Interest	Context	Methodology	Findings
Busanich, McGannon, Schinke [10]	2014	*n* = 1 female runner (age 34) *n* = 1 male endurance runner (age 19)	Experiences of DE and gendered constructions	Athletes in distance running	Narrative analysis	Athlete identity and desire for better performance led to dietary restriction, obsession with body size, and eventual injury/illness. Gender and stigma were evident in ED discussions
Thorpe and Clark [46]	2019	*n* = 12 female elite endurance athletes (age range: 30–38)	Sociocultural and biological pressures through experiences of LEA and REDs	Female athletes in endurance sports	Semi-structured interviews	Participants described a high-performance culture where a lean, toned body is seen as both aesthetically desirable and performance-enhancing, often involving restrictive eating and intense training. However, the resulting physiological consequences (e.g., menstrual disturbances, injury, LEA) revealed complex interactions between sociocultural ideals and biological responses
Langbein, Martin, et al. [47]	2021	*n* = 12 endurance athletes, (female *n* = 10, male *n* = 2; *M* age: 28.33 years (SD: 6.20))	Experiences of REDs	Athletes in endurance sports	Semi-structured interviews	All participants experienced physiological and psychological distress resulting from experiences with REDs
Langbein, Martin, et al. [48]	2022	*n* = 8 female endurance athletes (*M* age: 29.75 years (SD = 5.03))	Experience of recovery from REDs	Female athletes in endurance sports	Integrated methods, multiple case study	Participants described ongoing psychological tension in recovery, including loss of athletic identity, internalized performance expectations, and body image struggles. Recovery from RED-S was framed not as a linear process but as emotionally complex
Stewart, Allen, Kirkland [49]	2023	*n* = 2 female ultrarunners (ages not reported)	Role of coaches in managing REDs	Female athletes in ultrarunning	Semi-structured interviews	Psychological drivers, sport pressures, and the social environment play an important role in the development of REDs. Evident themes emerged: low self-worth, frustration, and the need for coach support
Is and Aydog [50]	2024	*n* = 1 female ultrarunner (age: 42)	REDs and Goldman’s dilemma	Female athlete in ultrarunning	Case report	Athlete was found to be positive for multiple stress fractures, low BMI, irregular menstruation, and was non-compliant to treatment of rest

* Athlete groupings and age data as reported in the study

#### Psychological distress and conflict

Athletes described feeling psychological distress from concerns such as the pressure to perform and/or look a certain way, fear of failure, managing athlete identity, and from managing the symptoms and consequences of REDs [10, 46–49]. One athlete revealed her experiences of pressure, stating: “I’ve always been a pretty driven perfectionist kind of person and just, and a people pleaser too so the thought that there was anything wrong with me that people didn’t like, then I had to fix that” [47]. Additionally, ultrarunners in one study discussed punishing themselves with exercise in response to failing to meet their performance expectations [10]. Elsewhere, an athlete described her worries about bone health: “‘I’ve had a lot of bone related injuries [.] Last year I was swimming and broke my hand […] my husband said “nobody breaks their hand swimming”, so it did make me wonder” [46].

Relatedly, other athletes in the reviewed studies exhibited psychological distress about knowing that they were engaging in behavior that was detrimental to their health and wellbeing yet not doing anything to ameliorate their experiences with REDs. Notably, a participant in one study involving male and female athletes affirmed: “It’s like you have two sides to your brain: the irrational and rational side, and it’s being able to decide which one is actually correct. You know the rational side is there but in moments where you’re really stressed out you just forget about it” [47]. Separately, athletes also expressed conflicts about recovery protocols “[I] just try and sneak in extra calories, but I still find it quite hard. Like if I was going on a massive run, I would. But when I’ve hardly broken out in a sweat just doing a few weights I’m like ‘really do I need to?’ [48].

In a case study of a 42-year-old ultramarathoner, psychological distress was not explicitly identified, though noncompliance to treatment protocols and the potential for resulting negative health outcomes was emphasized [50]. Researchers suggested that patients who present similarly should receive psychological evaluation as they might have the potential to engage in self-injurious behaviors [50].

#### Body image concerns

Appraisal of athletes’ physical appearance by external sources, together with their own fixations and fears about their physical being, were apparent as related to body image concerns in the reviewed qualitative studies [10, 46–49]. Notably, one athlete described her reaction to comments about her body and the subsequent effects on her fueling and activity choices: “I can really remember someone saying to me that they wanted my legs or something. And cause it had like come at a point where I’d just been like changing my diet and doing a bit more exercise, I thought “oh I can’t stop now’” [47]. In a different investigation, commonly-held beliefs about the correlation between certain body types and performance influenced athletes’ concerns, as demonstrated by an ultra-runner who stated: “Some people comment on your body when you get leaner, and they think that leaner is better” [49].

Internal convictions also played a role in how athletes feel about their bodies, with one conceding: “It’s just my own perspective […] I need to drop weight to perform well” [46]. Elsewhere, a participant in a separate study expressed feeling shame about their body after criticism: “I can remember actually someone a couple of years back saying to me, ‘well, you have a little fat around your thighs. I can show you some exercises how you can get rid of them’” [49]. Finally, one athlete recalled a shift in his career as body image drove him to reassess his routine: “I was like, ‘What could I change about myself to finally do well at the state meet? [.] I just became really conscious about my body” [10].

#### Caloric restriction

In response to both psychological distress and body image concerns, athletes reported restricting caloric intake in the studies represented in our review [10, 46–49]. Several athletes acknowledged engaging in food restriction to gain a sense of control inside and outside of sporting contexts. This was exemplified by perspectives from ultra-endurance participants, who affirmed “I like started trying to control what I ate and then it became a control thing” [47] and “if I have a particularly bad day and feel anxious, it could be anything, but I’ll end up eating less than I should” [48]. Another athlete acknowledged engaging in energy restriction after feeling pressure to improve his performance: “I guess what precipitated me to do the things that I did [.] eat super, super healthy – and work out twice as hard, and almost restrict (my)self […] was I thought that’s what elite level runners did. I thought that’s why people are running better than me – because they are more disciplined than me, they eat healthier than me” [10].

One athlete acknowledged the paradox of enhanced performance following a strict diet despite their physical concerns: “initially my performance was getting better, but it was a very fine line. My foot started to be sore, but I ignored it, carried on training hard and not looking after my body” [49]. In another source, all study participants acknowledged following stringent dietary practices, even if they realized that it could be detrimental to their overall health, but they were convinced that staying lean was necessary for optimal performance; one said: “For some reason, I have noticed [the desire to lose more weight] going on in my head and I’m aware of it and knowing it’s really really bad, but the leaner you get the less you want to eat” [46]. 

#### Physiological distress

Qualitative findings around REDs also indicated that the physiological implications of restriction and over exercise are wide-ranging. In this regard, across all studies, athletes reported experiencing various injuries and conditions, such as stress fractures and hormonal dysfunction [10, 46–49]. Some of the pain and injury came from athletes’ harmful misuse of physical activity: “Exercise then became punishment really. I just used to, well, whip myself with it. I think I’d got self-destruction down to a fine art by then, so it was basically self-harming” [47]. Furthermore, two ultrarunners from a different sample described self-injurious behaviors through more physical activity, less food, as well as alcohol use and/or performance-enhancing drugs [10].

In all studies, male and female athletes experienced physiological symptoms consistent with REDs. Athletes reported stress fractures, muscle and connective tissue injury, amenorrhea, hormonal imbalances, gastrointestinal issues, and other illnesses [46–50]. In certain cases, physiological distress helped athletes to reconsider their relationship with physical activity, as demonstrated by one athlete who attested: “I thought, right ‘do you want to be able to run a marathon, or do you want to go into hospital?’” [47]. However, this experience was not universal; for example, the athlete in the case study was willing to continue to train through severe injury [50].

## Discussion

The reviewed studies incorporated a mix of quantitative and qualitative research and there was heterogeneity between and among methodological designs. The quantitative findings provide clear evidence of risk and physiological sequelae for REDs [16, 22, 27, 34, 40–44]. Furthermore, the qualitative studies in the results give more context to the experiences of athletes, extending beyond statistical data provided in the quantitative studies. For us, the integration of the two is critical to an in-depth understanding of REDs in ultra-endurance sports.

### Physiology vs. psychology

The results in this review indicate that ultra-endurance races and associated high-volume training can create additional vulnerabilities for REDs, both physiological and psychological. Specifically, ultra-endurance athletes may struggle to meet in-activity carbohydrate demands during long distance events, as well as in everyday training efforts, which would be a minimum 60 g per 60 min for activities lasting longer than 2.5 h [22]. In particular, adequate carbohydrate intake may be critical to avoid stress hormone increases and the subsequent development of REDs and its related symptoms [6, 17]. Yet, ultra-endurance athletes may find it difficult to address these energy imbalances within the same day and/or correct within-day energy imbalances, potentially leading to a state of chronic carbohydrate depletion [6, 11]. Other physiological consequences can stem from inadequate caloric intake, such as stress fractures, muscle soreness, low sex hormones, and increased susceptibility to common colds (Mountjoy 2023). Bone stress injuries, for example, were reported in one-third (37.5%) of the female ultramarathon runners in one reviewed study [45]. Elsewhere, in another study in the results, male cyclists experienced low testosterone after a period of overtraining, which can also result in compromised bone mineral density with concomitant susceptibility to injury and possible sexual dysfunction [44]. Moreover, resulting injuries may force athletes to take time away from training and competition, potentially compromising performance and adding psychological strain [17].

This could be amplified by the unique psychological pressures that ultra-endurance athletes can experience, such as the tendency to equate finishing with self-worth, a heightened sense of body awareness, and a desire to withstand physical challenges [9]. Although psychological factors play a role in the development and consequences of REDs, their consideration was not consistently addressed across the reviewed studies [19]. Several (*n* = 5; 31.3%) of the qualitative studies did not explore psychological factors despite uncovering ED risks; that is, these studies did not explicitly focus on the psychological factors that might lead to ED and/or discussions about the need for mental health assessments. Certain quantitative studies in this review solely mentioned psychopathology that co-occurs or is concomitant with REDs (i.e., ED or EXD) without discussing psychological factors associated with these conditions [16, 40, 44]. Although we did not explicitly include EXD in our search strategy, as has been done elsewhere [26], samples involving ED/DE are often contextualized by discussions of addiction to physical activity. It is also important to note the well-established links between EXD and ultra-endurance sports despite a lack of recognition within the DSM-5/ICD-11 and clinical guidelines on symptoms, diagnosis, or treatment [26].

Viewing REDs as mainly a physiological concern can undermine the need for psychological care in certain athletes [28]. This is of critical importance, as physicians and other healthcare practitioners may not be universally aware of these complex conditions or how to diagnose, treat, or refer for specialized treatment [51]. The overlap in symptom presentation between mental disorders, such as depression and anxiety and REDs, can incur additional difficulties [52]. If assessed through the lens of REDs alone, athletes who experience psychiatric symptoms risk being dismissed as “dedicated” or “driven” when they would likely benefit from more comprehensive interventions [53].

Qualitative studies in our review revealed conflicting athlete attitudes, with participants frequently using phrases such as “broken” and “self-destruction” when recounting individual experiences [47, 48]. Excluding these psychological issues relating to body image, self-esteem, and performance pressures, underlined in the reviewed qualitative studies, may result in non-compliance, relapse, and/or further injury despite improved knowledge of nutritional best practices [27, 54]. The lack of scholarly research on these aspects may mean that treatment methods remain inconsistent; for example, past attempts at managing the syndrome by simply correcting the energy imbalance were found to be inadequate [54]. Likewise, weight training has been proposed as an intervention to improve muscle mass in athletes recovering from REDs, though an emphasis on additional exercise while in recovery may be determined counterproductive [17, 55].

General non-ultra-endurance specific literature seems to contain inconsistencies in the presentation of REDs and does not universally acknowledge that it is a syndrome with physical and mental health considerations [19, 56]. It is not clear if this is due to a reluctance to frame REDs as a mental disorder, notwithstanding recent work on the necessity of employing a psychological model when considering REDs [28]. Notably, this outlines the contributing risk components for REDs, such as body dissatisfaction, increased training volume, and self-esteem factors that ultimately lead to restriction, poor sleep, low mood, and anxiety [28]. A broadening of the topic and additional context may serve to fortify the pre-existing model of REDs in ultra-endurance sports and beyond; however, it could simultaneously demonstrate an even greater overlap to ED, with the only distinguishing feature being an attribution of maladaptive behaviors to an athletic ideal [28].

### Applicability of the reds model in ultra-endurance sports

Though REDs has become an increasingly important topic and brought more attention to possible hazards within unique sporting contexts, the utility of the term, alongside the breadth of foundational evidence has been debated [52, 56]. As described, REDs is said to result from LEA, but the analysis of energy availability has been considered unreliable due to methodological limitations, including dependence on self-reported dietary intake and potential inaccuracies in assessments of energy expenditure, such as indirect calorimetry [57, 58]. Resultant discussions of LEA may thus stem from observed symptoms and risk factors [52].

As our review substantiates, this could be particularly concerning in ultra-endurance sports, where energy intake during extended races and/or training for longer-distance and multi-day events will likely never meet expenditure [4]. Discussions of the REDs model include its applicability to both athletes and non-athletes, and those who intentionally or unintentionally under consume calories [4, 17]. Yet, it remains unclear whether the model is designed to investigate the potential personality characteristics that drive participation in ultra-endurance sports, which, for some athletes, would drive adherence to behaviors that remand the athlete to chronic LEA [59]. This is particularly important in the context of the findings in this review, with qualitative investigations showed that some athletes are using ultra-endurance sports to sublimate uncomfortable feelings and attitudes [10]. It would therefore be critical to understand other personality factors that may lead to unhealthy participation and any subsequent health consequences.

The REDs model recommends screening and return-to play tools, alongside other treatment recommendations, but does not specifically address the successful application of these protocols for the unique demographic considerations of many ultra-endurance athletes. Typically, the average ultra-endurance athletes is aged over 40 [60], not professional or elite, and might not have access to the recommended cadre of healthcare practitioners suggested in the REDs treatment guidelines [17]; discussion of the successful application of REDs protocols was not explicitly explored in any studies represented in our results [17, 61]. Equally, the Clinical Assessment Tool Version 2 (associated with the REDs model is complex and not designed to be self-administered by an independent athlete. Further, the CAT-2 does not utilize computational evidence of LEA; due to the potential for chronic underfueling in ultra-endurance athletes, this would be a critical metric to understand [52, 61]. Based on our search procedures, no such study exists with a primary focus on ultra-endurance athletes.

### Review strengths, limitations and future research directions

Our strategy for sourcing evidence for this review included incorporating both quantitative and qualitative studies to appropriately address available literature on this complex topic. This integration was a key strength. In general, there is a lack of scientific inquiry into ultra-endurance athletes and a resultant lack of concordance in definitions and methodologies. Consequently, we used a narrative review approach with the intention of capturing the spectrum of extant research. Through this, we identified wide heterogeneity in the assessment protocols among the literature represented here, as well as differences in the conceptualization of REDs itself.

Nevertheless, a non-systematic approach may engender concerns about biases in the selection of the materials in our results and reproducibility [62]. Equally, it is possible that predicating our eligibility criteria on subjective definitions of UES (i.e., training for UES events instead of including athletes who train similarly without a target event) may have omitted other relevant studies on REDs in these contexts. However, these sports are often difficult to conceptualize, and this approach was based on proposed descriptions elsewhere [1, 26]. Moreover, as this is a narrative review, we did not conduct a formal meta-synthesis or re-analysis of qualitative data, which may limit the depth and consistency of interpretive rigor. Though care was taken to select quotes representative of key themes, the potential for selection bias remains, and not all studies presented participant-level data in the same manner.

Our review revealed sizeable gaps in the evidence base that can be addressed through additional research [16, 50]. For example, future studies should consider whether the unique features of ultra-endurance sports lead to DE/ED and if there is a specific type of athlete who is drawn to UES as preliminary work demonstrates that these factors might play a role [63]. Concerningly, personality traits that are also associated with athletic success have also been shown to predispose ED risk and other maladaptive behaviours [63, 64]. Additional work could aim to uncover the links between these factors among ultra-endurance athletes and beyond.

Though the featured studies represented gendered diversity, there remains a lack of inquiry into males [65]. As use of the REDs model and subsequent educational efforts have resulted in male athletes recognizing risk [66], we hope that more initiatives are directed toward men. Furthermore, studies also focusing on non-binary, and transgender athletes who may be at increased risk for ED would be timely [67], since several UES events have recently created competitive categories for non-binary athletes [68].

Finally, initial research on treatment interventions in those athletes who are intentionally underfueling (whether amateur or elite) could be augmented by ED-based protocols [64]. As weight-related concerns may contribute to participation in ultra endurance sports [34], there may be a sizeable population of amateur athletes at-risk, even though they do not fit the stereotypical profile of an athlete vulnerable to experiencing ED. Accordingly, more research should be aimed at understanding whether the depth of knowledge of ED screening and treatment protocols is well-suited to serve the complex and multifactorial considerations of certain underfueling athletes.

## Conclusions

This review of quantitative and qualitative research on REDs and related conditions in UES revealed complex, multifactorial issues that might pose particular risks for ultra-endurance athletes. The results suggest that both physiological and psychological factors that may contribute to an athlete’s intention to under fuel and there are also physiological and psychological consequences to under fueling.

Critical to this discussion is the acknowledgement that there may be a wide range of ages, performance levels, and personality factors in the ultra-endurance population. This demographic heterogeneity may cause certain athletes to be overlooked, as they may not fit a stereotypical representation of what an at-risk athlete may “look like”. In addition to this, the unique characteristics of ultra- endurance sports may entail challenges and associated risks for REDs. It is crucial to emphasize that many of these concerns can be psychological. As this review demonstrates, body image concerns, performance pressure, and identity conflicts influence fueling behaviors, sometimes leading to DE or unhealthy exercise practices across the ultra endurance community It is also therefore important that individuals, as well as healthcare practitioners, have access to assessment tools that consider both physiological and psychological contributions to risk, irrespective of age, sex, or competition level.

Greater consideration of the extensive research on ED, which overlaps with REDs, could guide better and more holistic care for at-risk athletes. In the authors’ opinion, intentional LEA should be considered a signal that underlying psychopathology could be present, and this must be well-understood before an athlete will consent to necessary behavioral adjustments. As evidenced in this review, some athletes may still exhibit therapeutic non-compliance or be unwilling to modulate their training and nutrition strategies, even when they recognize health risks. Psychological resistance to change is a treatment barrier, and interventions that solely focus on the physiological aspects of REDs, such as caloric intake and rest, will likely be insufficient if these mental health components are neglected. If athletic performance is used as justification for improper fueling strategies, many health concerns could either be overlooked, considered “normal” in athlete populations, or simply set aside as athletes focus on an immediate race goal.

In sum, more comprehensive and integrated approaches to addressing REDs in UES requires collaborative schemes, incorporating both physiological and psychological health, as well as a commitment to recognizing challenges in a potentially diverse population. Existing research provides a strong foundation for understanding the complexities of energy availability and fueling in athletes; however, to sufficiently support ultra-endurance athletes, it may be necessary to broaden the scope of care, acknowledge the interrelationship between mind and body, and prioritize access to education and support for athletes at all levels. By corollary, the immediate and long-term health and performance can be improved, while engendering a culture of care that recognizes the human being and not just the athlete.

## Data Availability

No datasets were generated or analysed during the current study.
